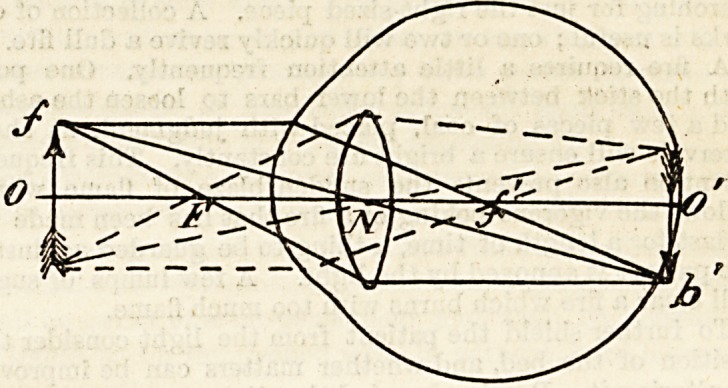# The Hospital. Nursing Section

**Published:** 1903-04-04

**Authors:** 


### The Hospital.

### Nursing Section.

Contributions for this Section of "THE HOSPITAL" should be addressed to the EDITOR, "THE HOSPITAL", NURSING SECTION, 28 & 29 Southampton Street, Strand, London, W.C.

No. 862.—VOL. XXXIV. SATURDAY, APRIL 4, 1903.

### Notes on News from the Nursing World.

### ROYAL VISIT TO IPSWICH.

THE Princess Christian, accompanied by her daughter, Princess Victoria, will visit Ipswich on May 9th in order to open the new nursing home of the Suffolk County Nursing Association, and to unveil a medallion of Queen Victoria. The home is in Lower Brook Street, and is practically a new building. There are nearly thirty bedrooms in the house, with many other spacious and convenient apartments, and it is surrounded by a pleasant lawn and numerous flower beds. The donor, a gentleman who does not wish his name to be disclosed, has not only purchased the house and garden, but has also furnished and fitted up the former in a very handsome manner. Purses of the amount of £3 and upwards may be presented to the Princess Christian at the opening ceremony.

### THE WOMENS' MEMORIAL TO QUEEN VICTORIA.

LADY DIMSDALE must be warmly congratulated upon the handsome amount which she has been able to hand to Lady Londonderry's Committee as the result of the City of London collection for the women's memorial to Queen Victoria, in connection with the Jubilee Institute. This sum, £3,888, is the largest contribution for the purpose by any county or borough committee in the United Kingdom, and, as may be imagined, it represents a very considerable expenditure of time and energy on the part of the popular wife of the late Lord Mayor of London, who at a Mansion House meeting last year delighted an influential gathering by the business-like manner in which she took the matter in hand.

### THE COUNTESS OF DUFFERIN'S FUND.

THE annual report of the organisation known as the Countess of Dufferin's Fund, which is also the National Association for Providing Female Medical Aid to the Women of India, has been issued. Among the interesting statements it contains may be mentioned the fact that there are now in India about 260 hospitals, wards, and dispensaries of various kinds for the treatment of women which are officered by women, most of them directly governed by or affiliated to the Association. Forty lady doctors of the first grade, 82 assistant surgeons, and 177 hospital assistants or practitioners of the third grade, are employed in the various Zenana hospitals and institutions. As to female students, there are now, including nurses and compounders, 498 women studying medicine in the various classes of the medical colleges and schools in the different Provinces. Hariot, Marchioness of Dufferin and Ava, expresses her deep regret that owing to illness, following on a time of great trial—the death of Lord Dufferin—she could not produce the report earlier, and adds that even now it was only through the kindness of Miss Heather-Bigg that she was able to prepare it. From the financial point of view it is noteworthy that Lady Curzon's Victoria Memorial Scholarships Fund for training Indian midwives now stands at seven lakhs of rupees, which brings the total investments of the Dufferin Fund to about 34 lakhs of rupees.

### FUNERAL OF MISS L. M. GORDON.

ON Thursday afternoon, last week, the remains of the late Miss L. M. Gordon were laid to rest in the pretty and sunny cemetery at Hove in the presence of many sorrowing mourners, who had assembled to pay their last tribute of respect and affection. Mr. Wainwright, the treasurer of St. Thomas's Hospital; Mr. Bonham-Carter, the secretary of the Nightingale Fund; Dr. Tate, Dr. Bourning, and other personal friends, including Miss Hamilton, the matron, and several members of the nursing staff were present. Of the many beautiful flowers sent, those bearing an inscription in the handwriting of Miss Florence Nightingale were placed on the coffin and lowered into the grave. A most impressive service was conducted by the Presbyterian minister, the Rev. Mr. Gordon.

### MEMORIAL SERVICE AT ST. THOMAS'S HOSPITAL.

WHILE the funeral of the late Miss L. M. Gordon was proceeding at Brighton on Thursday last week, a memorial service was held in the chapel at St. Thomas's Hospital. All the sisters and nurses who could leave the wards were present, as well as several members of the medical and surgical staff, and a large number of friends who entirely filled the front benches. The service consisted of the office for the Burial of the Dead, a few specially selected collects being substituted for the portion to be read at the grave. The hymn "On the Resurrection Morning" was sung by the nurses' choir, who also chanted the special psalms. The chaplain, the Rev. W. Weigall, conducted the service, and the lesson was read by the assistant chaplain, the Rev. R. Turner. At the close, while all remained standing, the organist played the Dead March from "Saul." Among the visitors was Miss Sidney Browne, matron-in-chief, Q.A.I.M.N.S. Every facility was given to the staff by the matron of St. Thomas's, Miss Hamilton, to attend the service. Miss Hamilton, who went to Brighton to be present at the funeral, was accompanied by some of the sisters and five of the nurses. Mrs. Swan, the assistant matron, who was with Miss Gordon at the last, remained at Hove until after the ceremony.

### THE FEMALE NURSES ON THE "MAINE."

Last week in the House of Commons Mr. Tennant asked the Secretary of State for War whether, in view of the fact that the American orderlies and male nurses on the hospital ship *Maine* had received the war medal for their service to our sick and wounded in South Africa and China, he would state why the five certificated American female nurses in charge had been refused the medal. Mr. Brodrick replied that "instructions were dispatched to the principal ordnance office at Woolwich on March 13th to issue these medals." This is only satisfactory so far as it goes. The American female nurses on the *Maine* ought to have received their medals at the same time as the American orderlies and male nurses, and we cannot understand why they did not.

### THE NEW SECRETARY OF THE BRITISH NURSES' ASSOCIATION.

Miss Annie Hobbs has been appointed secretary of the Royal British Nurses' Association in succession to Miss Leigh. She was trained at the West London Hospital, and has since enjoyed a varied and useful experience. She has had charge of a floor of the Establishment for Invalid Gentlemen in Harley Street, she has been night superintendent of the Women's Hospital in Soho, assistant to the superintendent of the Nurses' Co-operation, and secretary of the Auxiliary Nurses' Society. In each of these positions she showed considerable ability, and her intimate knowledge of the requirements of private nursing should be of practical assistance to her in the discharge of her new duties as principal official of an organisation of private nurses.

### DISCUSSION ON NURSING HOMES.

The members of the Royal British Nurses' Association discussed last week the question of nursing homes. The chair was taken by Miss Georgina Scott, and short papers, dealing with various aspects of the subject, were read. Miss Forrest dealt briefly with financial matters; Miss Titherington advanced a plea for small homes; Miss Mocatta advocated the public need of nursing homes, and Miss Annie Hobbs gave "the nurses' point of view." Miss Hobbs said that objections were frequently urged by nurses; for example, there were heavy trays to carry up and down stairs, and they "lived in their boxes," while it was no uncommon thing for the bathroom to be the general dressing-room for the nurses. There were dozens of homes in which a fully-trained nurse was scarcely to be found; they were frequently started with insufficient capital, and the salaries were too low to attract qualified women. In a few years the unqualified women employed at such places set up nursing homes of their own. "We hear much," she concluded, "about there being too many nurses, and about their shameful conduct. If these things could be rectified we should have a good deal less criticism of our most honourable profession." Miss McCall, in an energetic speech, urged the necessity of an operating theatre in every nursing home, of good salaries for the nurses, of employing only those fresh from the best hospitals, and no "probationers," and the placing of all homes, or, as she preferred to call them "private hospitals" under Government inspection. The discussion turned chiefly on the points raised by Miss McCall, and was sustained among others by Miss Blanch Trew, matron of the Royal Cornwall Infirmary, and Miss Young. A vote of thanks to the readers of the papers was proposed by Miss Trew and seconded by Miss White, late matron of the Wolverhampton and Staffordshire Infirmary.

### THE NURSING STAFF AT ST. MARY'S HOSPITAL.

It has been felt for some time past that the nursing staff at St. Mary's Hospital, Paddington, should be augmented. Owing partly to the increasing number of patients, and, more particularly, to the growing number of important operations, the pressure on the nurses has been steadily and continuously increasing, while it has been impossible, for lack of room, to add to the number. Now, however, with the near approach of the completion of the Clarence Memorial wing, the much-needed additions are within measurable distance of being made. The top stories of the extension in Praed Street will contain 120 rooms, which are arranged on the principle of giving each nurse a separate bedroom. It is probable that on account of the shape of the building, there will have to be a few double-bedded rooms, but only where it is impossible to arrange otherwise, and the majority of the nurses will certainly have separate sleeping accommodation. There will be at least three sitting-rooms, one of which will be a "quiet" room for reading, writing, etc. The dining-room will remain in the original building. Of course, the opening of the Annie Zung Ward in the new wing will considerably increase the work, and altogether it is anticipated that something like 35 nurses will be required to augment the present staff.

### THE NEW SUPERINTENDENT OF THE HYDERABAD STATE HOSPITAL.

Miss Brenda Hoare, who last year resigned her appointment in the Army Nursing Service Reserve and went to India for a few months' holiday, has been appointed lady superintendent of the Afzul Gungj, a State hospital of Hyderabad for natives. It contains from seventy to a hundred beds, and there are ten Eurasian nurses, who live in their own homes and come to work in the hospital every morning. The salary of the lady superintendent is 500 rupees per month, equal to about £350 a year, but no quarters are provided for her and there are no allowances of any kind. She is expected to visit the hospital every morning and evening.

### NURSES AND CHURCH MISSIONS.

Several hundred nurses availed themselves of an invitation sent out by the Chnrch Missionary Society to attend a conversazione on Wednesday afternoon last week. Invitations were addressed to all the London hospitals, and the nurses arrived at Salisbury Square to find a programme of music, tea, exhibitions of curiosities from foreign countries, and lectures on mission work prepared for them. One of the lecturers was a doctor from the Punjab, Mr. Martyn Clark, M.D., another was a clergyman from Palestine, the Rev. R. Elliott, and the third, a lady doctor, Miss Emmeline Stuart, M.B., from Persia. The collection of curios included some primitive-looking surgical instruments from China, Afghanistan, and Persia, models of native hospitals, objects made by lepers, etc. An excellent programme of music was rendered. There are now 33 fully-qualified nurses working in connection with the society abroad.

### "NURSING" UNDER AN URBAN DISTRICT COUNCIL

During the progress of the trial of a case last week, when a market gardener and his wife sought to recover damages from the Gillingham Urban District Council for the death of their little girl from small-pox, whom they contended lost her life through negligence, some curious evidence was brought forward. When the first case of small-pox was discovered, the council had the patient put into a stable 15 feet by 12 feet, where there was no chimney, and the smoke came out of the top of the door, which was left open. Furthermore, the "nurse" in charge was an old man, an ex-soldier, who stated that he carried out the instructions given him; but it is contended that medicine bottles and the remains of sucked lemons were allowed to lie around the stable, and that the refuse was not disinfected properly and was thrown on to the land instead of being dug into trenches. This the "nurse" indignantly denied, but the jury, without leaving the box, awarded the parents £250 damages. Perhaps the verdict may have the effect of impressing upon other urban district councils that, if they have no isolation hospital ready for such cases, they can at least secure the services of a trained nurse, who understands how to minimise the risk of infection.

### A NEW MATRON FOR THE CAMBRIDGE ISOLATION HOSPITAL.

It will be observed from our list of appointments to-day that the Cambridge Corporation have appointed a new matron for the Infectious Diseases Hospital. In view of the friction which has taken place lately at the institution, it may be an advantage to have a fresh head of the staff. Miss Wright, who comes from Goole to Cambridge, has had thirteen years' experience of the kind which should enable her to satisfactorily discharge her new duties, though it does not appear that she has spent any of the time at a general hospital or infirmary.

### MIDWIFERY INSTRUCTION AT BRENTFORD INFIRMARY.

The lying-in wards of the Brentford Union have just been removed from the workhouse and placed under the charge of the medical superintendent and the matron of the infirmary at Isleworth. In future, therefore, probationers, after completing their three years' general training in medical and surgical nursing and massage at the infirmary, will be able to take a course of midwifery instruction.

### SOMERSETSHIRE NURSES IN COUNCIL.

Another of the small but useful gatherings of nurses which have lately been held in Somersetshire to talk over matters of interest to members of the profession took place at Kingston Grange, near Taunton, last week, and there was a good muster. Miss M. Froy read a well-thought-out and very practical paper on the "Feeding of Infants," which was listened to with great attention, and afterwards discussed.

### A STRANGE DECISION AT ELLESMERE.

The Ellesmere Board of Guardians have come to a curious decision in the case of a nurse who refused, and still refuses, to comply with their order to submit to vaccination. Instead of deciding to dismiss this contumacious young woman, the Guardians, by a majority of one, have determined to test her capabilities for a further period of three months. The presumption is that if they find she is an excellent nurse, they will retain her in their service, at any rate until there is an outbreak of small-pox at the Ellesmere Workhouse, in which event she would doubtless be sent away in a hurry.

### NORTH LONDON NURSING ASSOCIATION.

At the annual meeting of the subscribers and friends of the North London Nursing Association, which was held in the Polytechnic, Holloway Road, an interesting report of the year's work was read and adopted. The working men in North London showed their appreciation of the services of the nurses by presenting a gift of £30. The total number of individuals nursed was 1,577 and the number of visits 32,577. In contrast with the substantial help rendered by the working men is the diminishing support from the churches and chapels of the large district served by the nurses. The total contribution from this source was only £54 16s. 8d. The congregations attending the churches and chapels of North London ought to be ashamed of themselves.

### NATIONAL POOR-LAW OFFICERS' ASSOCIATION.

At the annual meeting of the members of the National Poor-Law Officers' Association the new president, Mr. T. W. Mallam, reported the proceedings of the council in regard to the reports of the Departmental Committee on the Nursing of the Sick Poor in Workhouses, and stated that a special sub-committee had been appointed to go into the subject and thrash it out. It was urged by several speakers that other classes of officers were concerned in the question besides masters and matrons and nurses, and it was decided to make additions to the sub-committee.

### THE DISCIPLINE OF THE NURSE.

The American Society of Superintendents of Training Schools for Nurses recently discussed at Detroit the question of the discipline of the nurse. Miss Alice Twitchell, superintendent of the S. R. Smith Infirmary, Staten Island, read a paper on the subject, in which she urged the need of military discipline largely upon the ground that, "the women who take up nursing in this age seem, as a general thing, to imagine that they know much better how to manage the work than their superior officers." Miss Dock condemned the punishment of taking away a nurse's cap for the time being as a wrong thing, based on a wrong principle, "because it humiliates the nurse publicly and does not do any good." Miss Delano was hostile to the idea of trying to turn out women of the same mould "with exactly the same ideas, warranted to run a certain number of hours a day and sleep a certain number of hours a day, regardless of that woman's individuality," and yet she thought that many training schools in the States had for years worked on that line. Miss Allerton contended that no two nurses being alike in disposition, no two should be punished alike. Miss Gross argued in favour of controlling the pupils generally by love, but admitted that for a few nothing else than rigid discipline availed.

### The Nursing Outlook.

"From magnanimity, all fear above;
From nobler recompense, above applause,
Which owes to man's short outlook all its charm."

### AGE, AND OLD AGE.

Some little time ago Mrs. Charrington delivered at the Pharos Club a very interesting address on "The Woman of Forty," in which she maintained that that was the age of a woman's greatest power and best development. We are reminded of this by seeing an advertisement of the Auxilliary Nurses' Society, which is a Co-operative Association of middle-aged women, who, according to their own account, have combined to meet two needs. They say :—

"Considerable difficulty is frequently experienced by medical men in obtaining permanent nurses for prolonged and chronic cases, and to supply this want is one of the main objects of the Society.

"It aims also at meeting the need for a body of nurses qualified by their age and experience to undertake cases in which either on account of the nature of the malady, or of the circumstances of the patient, it is undesirable to employ the services of younger women. All the nurses on the staff have been very carefully selected with a view to their physical fitness for their duties as well as their nursing qualifications."

Now women are much more ready than men to give way and retire before advancing age; in the past, and in society, when looks were considered the great point in a woman and her one great outlet was the marriage market, there was probably some force in this; but the superstition that a woman is old at 40 has unfortunately remained till to-day with women who are in a perfectly different position. In nursing, for instance, where experience counts for so much, and where the stability of years fitly meets the responsibilities and cares of dwelling ever in the midst of sickness and of death, the woman of 40 is at her best. And in a profession which calls for no distinction or even remembrance of sex, it is much wiser to secure the middle-aged nurse in many cases. This is no stigma on the nurse; after a few months in hospital any false shame of this sort departs for ever; but the friends of private patients do not understand this always, and the conventions of this world are generally wisely founded on universal needs. Let us never give the enemy cause to blaspheme or to think evil; let the young nurse be as careful as is possible to hold herself above reproach.

A young doctor has been known to retire from an hysterical female case, and to advise that an elderly doctor should be consulted in his place, so that we are not suggesting to the nursing profession any greater care than that exercised in the medical profession. And so there is considerable room for the elderly nurse, and room for a protest against the view that a woman grows old sooner than a man. The politician of 40 is considered a boy; when he gets to 50 he may be listened to; and at 60 he may be placed in power. Lord Salisbury was over 70 when he retired, and his young nephew who now makes the old fogies shake their heads is aged 54. Richard Whiteing was nearly 60 before he took to novel writing, and produced his popular "No. 5 John Street." Mr. Herbert Spencer, who ranks as the greatest philosopher of the age, was 40 before he planned the "Synthetic Philosophy" on which his fame rests, and 70 before he completed it. We should like to see some woman of 40 just starting out on her career, instead of thinking, as she too often does, that the best of her life is over, and that there is only a rapid going down hill in front of her. And in the mere matter of years the woman outnumbers the man; she has a greater chance of longevity, and to begin to settle down to invalidism and old age at 50, when perhaps you have as many years ahead of you as behind you is a great mistake. There lives at Wellingborough an old lady, Mrs. Pendered, aged 99, who has all her faculties, and is intensely proud of the way she has retained her powers. If she had begun to give up interest in life and her relations at 60, as many a woman does, she would have had to endure 40 years of wearisome waiting and dreary do-nothingness, instead of being interested and helpful for all this time.

Let it be granted that after 60 the strain of private nursing—of constantly passing from one severe case to another, and ever struggling hand to hand with death—is not advisable; still there is no need to give up altogether. Such a society as the Auxilliary Nurses', which flourishes by providing elderly nurses to chronic cases is a proof of this. And there are many other paths open. There are the school nurses, who go round visiting the elementary schools, and who, therefore, have all their Saturdays and Sundays free, and all the many school holidays that the spoilt child of to-day insists on. This is very light work, and the unfortunate wee bairnies become very dear to the tender heart of the old-maid nurse, and in her sympathy with them she can remain young and happy and beloved for many a long year. And then there is the demand for nurses for crêches —nurses who are thoroughly trustworthy to replace the old-fashioned "motherly" and untrained woman, who failed to see the necessity to keep the babies clean and not to mix up their bottles, or put them in one another's cribs. Here, again, the hours are short, the work light, and the Sundays are free. No need to give other examples; only just one final word of protest that a woman is no older than she feels, and that the way to keep young is to regard 40 as the prime of life, and not to think of retiring at 60, but merely to seek some more suitable employment, and keep the heart and the hands young by using both.

### Lectures on Ophthalmic Nursing.

By A. S. Cobbledick, M.D., B.S.Lond., Senior Clinical Assistant Royal Eye Hospital, late House-Surgeon and Registrar, Royal Eye Hospital.

### LECTURE VII.—FUNCTIONS OF THE RETINA (*cont.*)—MOVEMENT OF THE EYEBALL—FORMATION OF THE RETINAL IMAGE—GENERAL HINTS ON NURSING.

However short a time a light stimulus may last, *e.g.* the time occupied by a flash of lightning which is practically instantaneous, the impression on the retina is continued for about an eighth of a second; the image so formed is termed an *after-image*.

These images may be positive or negative; all positive images are a facsimile of the object seen. As a rule negative after-images are the result of a very strong and prolonged stimulation. The light parts of the object appear dark, and the dark parts appear light in the after-image.

*The field of vision.*—It is a matter of everyday experience that whilst looking straight in front, we have a view, somewhat indistinct it is true, of objects on either side of us; this fact is due to the incidence of oblique light rays passing to the anterior half of the retina. The indistinctness of the objects at the periphery of the field of vision is due to the light rays falling on that part of the retina which is some distance from the macula lutea. When we require a very distinct view of an object, and also when we wish to examine the colour of an object, we look straight at it, so as to obtain an image of it on the macula, which is the point of clearest vision.

*Movements of the Eyeball.*—These are controlled by the extrinsic muscles, which by acting together in different combinations, render a very wide range of vision possible. The two eyes move in conjunction, and by no effort of the will can one eye alone be moved.

In looking, *e.g.* to the left, it is the left external rectus which moves the left eye, but the right internal rectus that moves the right eye, so that the external recti do not work together. It must be remembered, however, that in accommodation for near objects, convergence takes place, and the two internal recti do come into action together.

The chief movements are outwards, inwards, upwards, and downwards. Outward movement is accomplished by the external rectus. Inward movement is accomplished by the internal rectus. Upward movement is produced by two muscles, viz., the superior rectus and inferior oblique. Downward movement is the result of the action of the inferior rectus and superior oblique muscles. If the muscular movements of one eye are affected by disease, double vision (diplopia) results, and is due to the fact that the two images formed do not fall on parts of retina which correspond in the two eyes. This can be produced artificially by pressing on one eye in such a manner as to prevent it from following the movements of the other.

Abnormal movements will be discussed in dealing with diseases of the eye, *e.g.* the marked protrusion in Graves's disease, and nystagmus, which is a rhythmical movement of the eyeballs.

*Elementary Optics.*—Only those points will be dealt with which are necessary to explain the formation of an image on the retina.

The eye may be looked upon as a camera: the retina corresponds to the sensitive plate, the iris forms the diaphragm, which shuts out all unnecessary rays of light, and the cornea, lens, and the two humours are the refracting media. The chief optical properties of this camera are:—

1. The curvature of the surface.
2. The refractive power of the media.

The only rays of light passing from the air to the retina which are not deviated from their straight course, *i.e.*, not refracted, are those which pass through uhe centre of the system or optic axis, O O′ and those which meet at N—the nodal point which is the centre of the sphere. All other rays, *e.g.* F F′ are deviated or refracted to meet at F′, which is the principal posterior focus.

Another important point on the optical axis is F, the principal anterior focus, for all rays of light passing through it to the convex surface become refracted so as to pass into the eye parallel to the optic axis (O O′), *e.g.* Fbb′.

Bearing these points in mind, it is easy to see by the following diagram how the retinal image is formed.

The diagram shows that the image formed on the retina is inverted; nevertheless, objects are not seen inverted as the higher centres in the brain reinvert the image.

### General Hints on Ophthalmic Nursing.

The essentials for the making of a good nurse have been already mentioned. In regard to ophthalmic nursing, which to a large extent is surgical, cleanliness, attention to detail, and patience, are of primary importance.

Cleanliness of garb is of course necessary, but special attention must be given to the hands. The space beneath the nails is capable of becoming a hotbed of infection, although *apparently* it may be quite clean; therefore keep the nails short and well trimmed, and so minimise this possibility as much as possible.

For your own safety never disregard cracks around the nails or scratches about the hands, especially if you are likely to come in contact with septic cases, *i.e.*, cases where there is a purulent discharge from the eyes; always protect such abrasions with a small collodion dressing; through ignoring this small precaution many nurses and medical men have suffered unnecessarily and even lost their lives. The hands should be kept thoroughly clean with carbolic soap and hot water, *before* and *after* attending to a case. If the fingers come in contact with any purulent discharge they should be immersed in a strong antiseptic lotion, *e.g.* carbolic acid 1 in 20 of water, and then well cleansed with soap and hot water. Excepting under such circumstances, it is well not to use very strong antiseptics for the hands, as they act on the skin in such a manner that desquamation occurs over a period of some days, these scaly particles render the hands rough, more readily soiled than usual, and they may be a source of infection by falling off whilst bathing or dressing an eye. It must be remembered that the eye is peculiarly susceptible to septic infection, more especially after operations which involve an incision of the eyeball and a communication between the conjunctival sac and the interior of the eye.

Other points, which might *not* be thought of importance, are attention to the hair and teeth; there is no doubt that some inexplicable cases of infection can be traced to dandriff falling from the head of surgeon or nurse, or through placing an instrument in an unclean mouth when both hands are in use.

### How to Make Up a Fire and Keep it Going Noiselessly.

### EXAMINATION QUESTIONS FOR NURSES.

The question was as follows:—

1. How would you proceed to make up a fire and to keep it going noiselessly in the room of a patient sensitive to sound?

2. How would you prevent the light from it annoying the invalid?

### First Prize.

To make up and keep going a fire as noiselessly as possible I should dispense with all fire-irons, using instead a housemaid's glove for tongs, a stick for a poker, and a child's wooden spade for taking up the ashes. If I could obtain such a thing, I should place a zinc tray, thickly sifted over with sand, under the fire to catch the falling cinders. This would deaden the sound and could be carried out of the room for emptying. Coals should be kept in a wooden box —one with a division is best. It should be filled with selected pieces of coal; no dust or slack, quite small lumps one side, rather larger in the other, so that there need be no searching for just the right-sized piece. A collection of old corks is useful; one or two will quickly revive a dull fire.

A fire requires a little attention frequently. One poke with the stick between the lower bars to loosen the ashes, and a few pieces of coal, placed with judgment, at short intervals will ensure a bright fire constantly. This frequent attention also prevents the sudden blaze of flame which follows the vigorous poking of a fire that has been made up to last for a length of time, a thing to be guarded against if the patient is annoyed by the light. A few lumps of sugar will clear a fire which burns with too much flame.

To further shield the patient from the light consider the position of the bed, and whether matters can be improved by altering it. Bearing in mind that light from the window falling directly on the patient's face will be more annoying than firelight, and that a current of air flows from the window to the fireplace. If the bed is old-fashioned with a high wooden foot-piece, and is opposite the fire, the foot-piece makes a good screen. Otherwise a screen, small clothes-horse or towel-horse, covered with dark material, can be arranged between the patient and the bed. Shadows and lights on the ceiling, disturbing to delirious patients, can be obviated by a large frame covered with black sateen or Italian cloth, resting at one side on the mantelpiece and supported in a horizontal position by cords attached to the two opposite corners and to hooks or nails in the wall about 18 inches or 2 feet above the mantelpiece. It will be sufficiently high to prevent any difficulty in making up the fire beneath it. This used with the screen will effectually darken the room.

If the fire has to be laid, or re-lighted, I should use shavings or tow instead of rustling paper, and make sure that the wood was thoroughly dried, also I should avoid using large pieces of wood as these sometimes burst asunder with a loud report as the heat expands the air in the cavities. Damaris.

### Second Prize.

To make up a fire and keep it going noiselessly I should first remove all fire-irons, with the exception of a short poker or wooden stick of some kind, which I should keep lying flat inside grate.

Then I should strew sand to the thickness of half an inch all over hearth to deaden the sound of cinders falling.

The coal-bucket should be prepared outside room, each lump being wrapped separately in brown paper. I should keep an old loose pair of gloves and wear when making-up fire.

Cinders, slightly damped, are useful to keep a fire in when not much heat is required, and these should be prepared and placed in a sheet of brown paper, so that the paper and all could be lifted on when required.

To keep light from flickering and annoying the invalid, I should get a dark blanket or winter curtain and fasten one side to mantelshelf by means of weights, heavy books, or tacks driven into chimney-piece, letting a good bit hang down on either side. Then two chairs should be placed facing fire, at a distance of about four feet, and the blanket stretched to this and fastened over the backs of each chair with big safety pins. Do not fasten cloth at extreme edge, but allow some to fall on either side.

This plan will keep fire from flickering on ceiling and walls, and yet will not prevent the heat from coming out into room.

In infectious cases the same plan could be carried out in brown paper with little more difficulty.

A small screen or chair, with dark cloth or brown paper fastened to back, serves to prevent direct light from fire or lamp annoying patient, or if bed is opposite fire, the same could be fixed to rail of bed. "Peggy."

### The Prize Winners.

"Damaris" comes out first, because she mentions three essential things: a layer of sand under the grate to deaden the sound of falling cinders; the arrangement of the fuel in two different heaps, so that no hunting need take place, and the prevention of the flickering light striking on the ceiling. The chief defect of her paper is that she does not mention the obvious advantage of wrapping the lumps of coal in paper and her arrangement against the flickering light though very ingenious is a little too complicated.

"Peggy," the winner of second prize, writes a very good paper and would have beaten "Damaris," but alas! she is too deeply attached to brown paper. A most pernicious article to use in an invalid's fire, as it gives forth a pungent and suffocating smell. Her answer is, however, excellent in all other particulars.

### Honourable Mention.

This is gained by "Nurse C. L. Brooks," "Paul," "S.," and "Doubtful."

### Question for April.

How would you feed a patient suffering from enteric fever, whilst the nourishment ordered by the doctor is to be strictly liquid? N.B.—The question supposes that the medical man in attendance gives only general orders and leaves the nurse to exercise her discretion.

The Examiner.

### Rules.

The competition is open to all. Answers must not exceed 500 words, and be written on one side of the paper only. The pseudonym, as well as the proper name and address, must be written on the *same* paper, and *not on a separate sheet*. Papers may be sent in for fifteen days only from the day of the publication of the question. All illustrations strictly prohibited. Failure to comply with these rules will disqualify the candidate for competition. Prizes will be awarded for the two best answers. Papers to be sent to "The Editor," with "Examination" written on the left-hand corner of the envelope.

N.B.—The decision of the examiners is final, and no correspondence on the subject can be entertained.

In addition to two prizes honourable mention cards will be awarded to those who have sent in exceptionally good papers.

### The Midwives Act, 1902: the Legal Aspect.

On July 31st, 1902, an Act was passed entitled "An Act to secure the better training of Midwives and to regulate their practice." This statute comes into force on April 1st, 1903. The following are its chief provisions:—

From and after April 1st, 1905, any woman who not being certified under this Act shall take or use the name or title of midwife shall be liable on summary conviction to a fine of £5.

From and after April 1st, 1910, no woman shall habitually and for gain attend women in childbirth, otherwise than under the direction of a qualified medical practitioner, unless she be certified under this Act. Any woman so acting without being certified under this Act is liable on summary conviction to a fine of £10, provided this section shall not apply to legally qualified medical practitioners, or to anyone rendering assistance in a case of emergency. No woman certified under this Act shall employ an uncertified person as her substitute. The certificate under this Act shall not confer upon any woman any right or title to be registered under the Medical Acts, or to assume any name, title, or designation implying that she is by law recognised as a medical practitioner, or that she is authorised to grant any medical certificate, or any certificate of death or of still-birth, or to undertake the charge of cases of abnormality or disease in connection with parturition.

Any woman who within two years from the date of this Act coming into operation claims to be certified under this Act shall be so certified, provided she holds a certificate in midwifery from the Royal College of Physicians of Ireland, or from the Obstetrical Society of London, or the Coombe Lying-in Hospital and Guinness' Dispensary, or the Rotunda Hospital for the relief of the poor lying-in women of Dublin, or such other certificate as may be approved by the Central Midwives Board, or produces evidence, satisfactory to the Board, that at the passing of this Act she had been for at least one year in *bonâ fide* practice as a midwife, and that she bears a good character.

On the passing of this Act the Lord President of the Privy Council shall take steps to secure the formation of a Central Midwives Board, which shall consist of—(1) four registered medical practitioners, one to be appointed by the Royal College of Physicians of London, one by the Royal College of Surgeons of England, one by the Society of Apothecaries, and one by the Incorporated Midwives Institute; (2) two persons (one of whom shall be a woman) to be appointed for terms of three years by the Lord President of the Council; and (3) one person to be appointed for a term of three years by the Association of County Councils, one person to be appointed for a term of three years by the Queen Victoria's Jubilee Institute for Nurses, and one person to be appointed for a term of three years by the Royal British Nurses' Association.

The duties and powers of the board shall (*inter alia*) be as follows:—

To frame rules regulating the course of training, supervising, and restricting within due limits the practice of midwives, deciding the conditions under which midwives may be suspended from practice, to decide upon the removal from the roll of the name of any midwife for disobeying the rules and regulations, and to issue and cancel certificates. Rules framed under this section shall be valid only if approved by the Privy Council; and the Privy Council, before approving any such rules, shall take into consideration any representations which the General Medical Council may make with respect thereto.

Any woman thinking herself aggrieved by any decision of the Central Midwives Board removing her name from the roll of midwives may appeal therefrom to the High Court of Justice within three months after the notification of such decision to her; but no further appeal shall be allowed.

Every council of a county or county borough throughout England and Wales shall, on the commencement of this Act, be the local supervising authority over midwives within the area of the said county or county borough. It shall be the duty of the local supervising authority (1) to exercise general supervision over all midwives practising within their area in accordance with the rules to be laid down under this Act; (2) to investigate charges of malpractice, negligence, or misconduct on the part of any midwife practising within their area; and should a *prima facie* case be established, to report the same to the Central Midwives Board; (3) to suspend any midwife from practice, in accordance with the rules under this Act, if such suspension appears necessary in order to prevent the spread of infection; (4) to report at once to the said Board the name of any midwife practising in their area convicted of any offence.

Every woman certified under this Act shall, before holding herself out as a practising midwife or commencing to practise as a midwife in any area, give notice in writing of her intention so to do to the local supervising authority, or to the body to whom for the time being the powers and duties of the local supervising authority shall have been delegated under this Act, and shall give a like notice in the month of January in every year thereafter during which she continues to practise in such area. And if any woman omits to give the said notices or any of them, or knowingly or wilfully makes or causes or procures any other person to make any false statement in any such notice she shall on summary conviction be liable to a fine not exceeding £5.

Any woman who procures or attempts to procure a certificate under this Act by making or producing, or causing to be made or produced, any false and fraudulent declaration, certificate, or representation, either in writing or otherwise, shall be guilty of a misdemeanour, and shall, on conviction, be liable to be imprisoned with or without hard labour for twelve months.

Any person wilfully making or causing to be made any falsification in any matter relating to the roll of midwives shall be guilty of a misdemeanour, and shall be liable to be imprisoned with or without hard labour for twelve months.

Where any woman deems herself aggrieved by any determination of any court of summary jurisdiction under this Act, such woman may appeal to the Court of Quarter Sessions.

This Act does not extend to Scotland or Ireland. The General Medical Council shall act by the English Branch Council, which for all purposes of this Act shall occupy the place of the General Medical Council.

### Presentations.

Royal Portsmouth Hospital.—Miss Hettie Shorto was presented with a handsome silver card-case by the nurses belonging to the private staff of the Royal Portsmouth Hospital on relinquishing her appointment as assistant matron.

Sunderland Nursing Institute.—Mrs. Marriner, who, in consequence of failing health, has resigned the post of matron of Sunderland Nursing Institute, has been presented by the committee with a beautifully bound volume of "Tennyson." She has also received from the nurses silver-mounted initialled toilet-sets with inscription engraved on mirror, a carriage-clock, an inkstand, a tea-kettle, a teapot, an afternoon tea-set, embroidered tray-cloths, and many other tokens of esteem.

### Everybody's Opinion.

### THE APOTHECARIES' HALL CERTIFICATE.

"One who Wishes to Know" writes: Will Lady Dispenser kindly give particulars of study taken up, how long necessary, and fees to qualify as a dispenser.

### THE PENSION FUND MEETING.

"A Matron" writes: I think that the sincere thanks of all the policy-holders in the Royal National Pension Fund for Nurses who were unable to attend the annual meeting are due to Sir Henry Burdett and the secretary, Mr. Dick, for the highly satisfactory statements made with regard to their business and reported in The Hospital. The account is so plain and full that anyone can understand the present state of affairs, and it must be a real comfort to nurses to know that their savings are so carefully invested and looked after, and that they have the advantage of the valuable time and advice of business men second to none in the kingdom.

### INTERFERENCE BY THE WORKHOUSE MASTER.

"A. F. C." writes: At our infirmary we are subjected to the perpetual interference of the workhouse master, who considers himself a better judge of the linen required to work the wards than the nurses who are in charge, and therefore refuses necessaries to them. No nurse trained in a general hospital ever stays here, and we cannot think that the Guardians are unaware of the state of affairs, if only because of the frequent "resignations," and also because one of their number spends a considerable portion of his time in the infirmary. Why cannot the master of the workhouse be made to keep his position; and why cannot we have as a head a lady trained in a general hospital, and not, as too often happens, an untrained person who has been promoted from midwife upwards?

### WARWICK GUARDIANS AND UNTRAINED NURSES.

"The Matron of Warwick Workhouse" writes: Your note in the edition of the Nursing Section of The Hospital dated March 21st about the meeting of the Warwick Board of Guardians is somewhat misleading. The Board did not object to the employment of fully-trained nurses at an adequate salary, which is proved by the fact that at a meeting held subsequently it was unanimously resolved to employ such nurses. The reason why the report was referred back to the committee was principally because the members of the committee were not agreed among themselves. Another point which cropped up was the consideration of the position of the matron of the Workhouse under the new nursing order, which it is presumed will shortly be issued. If the report of the Departmental Committee on Nursing in Workhouses is embodied in an order, in all places where a superintendent nurse is appointed the matron of the Workhouse loses her authority in the Infirmary. As this increases the status of the superintendent nurses and correspondingly decreases that of the matron it is naturally strongly objected to by most matrons and especially trained matrons. Take my case for instance. I was fully trained at University College Hospital, London, and besides holding other nursing appointments have been a matron for the last twelve years. Naturally I take very great interest in all that appertains to Infirmary work, and I feel that this new order will deal very unjustly with me if a superintendent nurse is appointed, and I am entirely shut out from the infirmary. I think anyone who understands institution work will agree with me when I say that it is better to have one competent female head of the whole institution than two, as being more conducive to harmony and smooth working. Too many cooks spoil the broth. If I may refer to another point in the report of the departmental committee, why should the superintendent nurse be compelled to hold a certificate for midwifery? This means that many capable general nurses will not be eligible for the office as a large proportion of them do not hold midwifery certificates. If one nurse on the staff is competent in this branch of nursing, surely this should be sufficient. Personally, I think that the guardians should have more discretionary power in these appointments, and not be bound too hard and fast by official orders, as the conditions vary very much in different workhouses. I trust that the Local Government Board will give the report further consideration before issuing an order.

### TRAVEL NOTES AND QUERIES.

Pressure on Space (To our Correspondents).—I greatly regret that owing to lack of room last week four answers to correspondents were unavoidably kept back.

Italian and Swiss Trips (Natt and Others).—Will all correspondents kindly notice that the two semi-private trips as above are no longer available. The numbers are made up and further applications are useless.

Switzerland viâ Neuchâtel (Sister Maud).—It is rather a cross-country journey that you propose making, but certainly it can be done. I think you can get a circular ticket through Gaze and Sons. The route would be viâ Dijon, and after you left Grandson (a truly charming spot) I believe there is a diligence service that would take you across to where you next want to stop. I will find this out for you. Look in this column next week. Are you good travellers? Would the break in Paris be sufficient, or do you need another before Neuchâtel?

Swiss Holiday (Jungfrau).—At Meiringen, the Croix Blanche the Post, and the Hôtel de l'Ours will take you from 5 francs. The cheapest route to Grindelwald is viâ Southampton and Havre, single, second class, £3 4s. 3d., return £5 6s. 4d. This is only to Interlaken. You book on to Grindelwald, 2s. 8d. Write to Henry Gaze and Sons at 53 Queen Victoria Street, E.C., using my name. Tell him what you want, and say you wish to break the journey at Meiringen, and for how long. I do not advise your walking over the Great Scheidegg; you would want a guide unless you go with an experienced mountain traveller, and it is a very long distance for a woman out of training. I do not know what note of mine you are referring to, so cannot answer about Berne.

Belgium in July (St. Patrick).—No, it is very unlikely to be too hot. There are plenty of pictures in Brussels and Antwerp. As to books, read Motley's "Rise of the Dutch Republic" and "Through the Ardennes," K. Macquoid. Do not try to take rooms, and Cook's coupons I do not much like except for very helpless travellers. Tell me how much you can spend each and how long you wish to be abroad and I will advise you as to hotels. You would like Dinant on the Meuse if the weather became very hot. Articles of mine on all the places you wish to hear about are in The Hospital for January 21st, September 2nd, October 21st and 28th, 1899, and in August 3rd and 10th, 1901.

Passage to Canada (Erin).—Second class on all the large ocean steamers is quite comfortable and you need have no misgivings. I am posting you a list of the different lines. The line I have marked is popular and most berths are booked up to early in May.

Belluno to Cortina (Royal Oak).—I find that there is no probability of diligences running nearly so early as you mention, but it depends greatly on the season, so that it is impossible to state with certainty until considerably later.

Caudebec or Rouen (Doubtful).—Caudebec is about 35 miles from Rouen because of the windings of the line. You certainly could visit Rouen in that way, but several visits which would be necessary would come to the same thing as hotel expenses for, say two days. The best and cheapest guide book is Black's "North France West Half," price 5s.

Boulogne (District Nurse).—Second class return £1 18s. 4d., third class return £1 5s. 2d. The latter is quite unobjectionable and the journey is very short. Write to Madame Auberlique, "Les Mauves," Wimereux, close to Dieppe, but her house is nearly always full. In Boulogne itself you would get good accommodation for 7 francs per day at the Hôtel des Bains de Mer. It is not a cheap place because it is overrun with English. Your journey (if third class) would be £1 5s. 2d., and 15 days' accommodation at 7 francs would be £4 4s., then you must reckon up your tips and excursions. Belgium would be cheaper.

### Appointments.

Afzul-Gungj Hospital, Hyderabad, Deccan.—Miss Brenda M. Hoare has been appointed lady superintendent. She was trained at the Royal Alexandra Hospital, Rhyl, and St. Thomas's Hospital, London. She has also been staff nurse at Nottingham General Hospital, and was lately a member of the Army Nursing Service Reserve.

Braintree Union Infirmary.—Miss Isabella Scott has been appointed assistant superintendent. She was trained at Plumstead Infirmary.

Borough of Cambridge Infectious Diseases Hospital.—Miss J. Wright has been appointed matron. She was trained at the Borough Sanatorium, Brighton, and has been nurse at Ashton-in-Makerfield Isolation Hospital, charge nurse at Swindon and District Isolation Hospital, and matron of Goole Sanatorium.

Cumberland Infirmary, Carlisle.—Miss Annie Moir has been appointed sister. She was trained at Beckett Hospital, Barnsley, and has been in succession night sister and sister of male medical and surgical wards at the North Devon Infirmary, Barnstaple.

Derby Union Infirmary.—Miss Marianne Frost has been appointed staff nurse. She was trained at St. Joseph's Hospital, Chiswick, and has been assistant nurse at the Union Infirmary, Cranbrook; the Union Infirmary, Hereford; and the Union Infirmary, Burton-on-Trent.

Epsom Union Infirmary.—Miss W. Redmayne has been appointed charge nurse. She was trained at Mile End Infirmary, and has since been head nurse at Romsey Union Infirmary and charge nurse at South Shields Infirmary.

General Hospital, Birmingham. — Miss Elizabeth Dockrell has been appointed sister of the battery-room and masseuse. She was trained at the Brentford Union Infirmary, Isleworth, for three years, and while there she obtained the certificate of the Incorporated Society of Trained Masseuses. She has since been for a few months staff nurse at the General Hospital, Birmingham.

General Hospital, Weston-super-Mare.—Miss Jessie H. Handy has been appointed staff nurse. She was trained at Pembrokeshire Infirmary, Haverfordwest, and has since been staff nurse at the City of London Hospital for Diseases of the Chest, Victoria Park, E.

Johnstone Combination Hospital, Renfrewshire.—Miss Lillian Muir has been appointed matron. She was trained at the City Fever Hospital, Edinburgh, and the Western Infirmary, Glasgow. She has since been assistant matron at Perth District Asylum, matron of a private asylum in Dublin, and sister at the Western Infirmary, Glasgow.

Kingston Hill Union Infirmary.—Miss Mary Barrett and Miss A. E. Tomkins have been appointed sisters. Miss Barrett was trained at the Middlesex Hospital, and she has since been on the nursing staff at the Tunbridge Wells General Hospital, and has done private nursing at Tunbridge Wells on her own account. Miss Tomkins was trained at Isleworth Infirmary, and for the last three years has done private nursing in Exeter and abroad.

Medical and Surgical Home, Sheffield.—Miss Annie Nelson has been appointed staff nurse. She has just completed her three years' training at the Brentford Union Infirmary, Isleworth.

Much Wenlock Hospital.—Miss Lydia Baskett and Miss Mildred Hodges have been appointed sisters. Miss Baskett was trained at King's College Hospital, London, and has since been night superintendent at the Royal Hospital for Diseases of the Chest, City Road, E.C, and home sister at Bristol Royal Infirmary. Miss Hodges was trained at Edinburgh Royal Infirmary. She has since been Queen's Nurse, has done private work, and has been night superintendent at Salop Infirmary, Shrewsbury.

Oldham Union Infirmary.—Miss Matallis Healey has been appointed superintendent nurse. She was trained at Oldham Union Infirmary.

Pontypridd Union Infirmary.—Miss Rose Fisher has been appointed superintendent nurse. She was trained at Lambeth Infirmary, and was afterwards staff nurse in the same institution. She has also been superintendent nurse at Newton Abbot Workhouse Infirmary, and superintendent nurse at South Grove Workhouse Infirmary.

Rotherham Hospital and Dispensary.—Miss E. Singer has been appointed sister. She was trained at the Western Infirmary, Glasgow, and has since been charge nurse at the Lady Hozier Convalescent Home, Lanark, sister-in-charge of the scarlet fever ward at the Isolation Hospital, Glasgow, and sister at the Western Infirmary, Glasgow.

Royal Portsmouth Hospital.—Miss Elizabeth Shackleford has been appointed assistant matron. She was trained at the Royal Infirmary, Liverpool, and Manchester Children's Hospital, Pendlebury. She has been sister at Brompton Chest Hospital and assistant matron at Victoria Park Chest Hospital, and has been attached to district nursing homes in Liverpool.

Royal Westminster Ophthalmic Hospital.—Miss H. Shorto has been appointed matron. She was trained at the Royal Surrey County Hospital, Guildford, and has since been assistant matron, and in charge of the Nurses' Home, at the Royal Hospital, Portsmouth.

Sick Asylum, Cleveland Street, London.—Miss Edith Russell and Miss Elizabeth M. Lovegrove have been appointed charge nurses. Miss Russell was trained at Lambeth Infirmary, and has done private nursing at Harrow-on-the-Hill. She holds the L.O.S. certificate. Miss Lovegrove was trained at University College Hospital, London, and has been staff nurse at Brompton Hospital, sister at Lambeth Infirmary, and charge nurse at St. Saviour's Hospital, Osnaburgh Street, London.

St. William's Hospital for Infectious Diseases, Rochester.—Miss E. L. Hawkes has been appointed matron. She was trained at St. George's Hospital, London, and has since been charge nurse, night superintendent, and housekeeper at the Fountain Hospital, Tooting, S.W.

Swansea General and Eye Hospital.—Miss Gertrude Holmes has been appointed assistant matron, and Miss Eva Margaret Hunter and Miss Mary Jane Thomas have been appointed sisters. Miss Holmes was trained at the Queen's Hospital, Birmingham, where she was afterwards sister and night superintendent. She has also been sister at the Ear and Throat Hospital, Birmingham, and sister at Swansea General and Eye Hospital. Miss Hunter was trained at Birmingham General Hospital. Miss Thomas was trained at Swansea Hospital, and has been sister at Merthyr Tydfil General Hospital.

Urmston Cottage Hospital, Manchester.—Miss Mary E. Froggatt has been appointed matron. She was trained at the Royal Hospital, Salford, and has since been sister in the same institution. She has also done private and district nursing.

Victoria Hospital, Blackpool. — Miss E. Lewis has been appointed sister. She was trained at the Royal Infirmary, Liverpool, and has since been sister at the Corbett Hospital, Stourbridge. She has also done private nursing.

West Norfolk and Lynn Hospital.—Miss L. Varley has been appointed charge nurse. She was trained at the District Infirmary, Ashton-under-Lyne.

### Echoes from the Outside World.

### Movements of Royalty.

On Monday the Queen left London for Copenhagen, the King and the Prince of Wales being present to see her Majesty off at the station. Prince Charles of Denmark accompanied her on the journey. The Queen looked remarkably well, and wore a dark travelling dress with a sable boa, and had a spray of Malmaison carnations in her dress. Although the weather was rainy, and it was evident that a gale was brewing, the Queen would not postpone her departure from Dover, and reached Calais safely, although the seas broke over the vessel with more or less severity all the ay. The Queen travelled right through to Copenhagen, which she reached on Tuesday evening.

The King left London the same afternoon for Portsmouth on his way to Lisbon. The departure was attended with some ceremony, and his Majesty wore the undress uniform of an Admiral of the Fleet. Amongst those who came to bid farewell to the King were the Duke of Connaught and Lord Roberts. Upon arriving at Portsmouth the weather report was so adverse that it was decided to spend the night in harbour. The Royal yacht left early on Tuesday morning.

### Scottish and Irish Visits.

The King and Queen, accompanied by Princess Victoria, have announced that they intend to visit Scotland in May, arriving on Monday the 11th and leaving the following Friday. During the period they will reside at Dalkeith Palace, which the Duke and Duchess of Buccleuch will vacate for the five days, and a Court and Levee will be held at Holyrood, whilst one day will be employed in a visit to Glasgow. It is officially announced that the King and Queen will visit Ireland in July or August. At present, of course, no details are forthcoming, but the intimation has given the Irish people intense satisfaction.

### Return of the Duke and Duchess of Connaught.

After an absence of about four months the Duke and Duchess of Connaught have returned to England. Before their arrival at Portsmouth on Friday last, the Duke, who sailed in the battleship *Renown*, handed to Captain Farquhar a memorandum which expressed the satisfaction of himself and the Duchess with the smartness with which the ship's company had worked throughout the voyage, and thanked them for the way in which they had discharged the extra duties devolving upon them in consequence of their Royal Highnesses' presence on board. At Portsmouth the Duke received an address from the Mayor and Corporation, and in the course of his reply made a brief reference to his visit to India, saying he would have great pleasure in reporting to the King the deep loyalty of His Majesty's Indian subjects. At the Harbour Station the special train to London was stopped in order that the Duke might inspect a guard of the Rifle Brigade, and at Victoria Station their Royal Highnesses were received by a distinguished company, including the Prince and Princess of Wales and the Princesses of Connaught. As the Royal carriages emerged into the crowded streets on Friday afternoon the people cheered vigorously, and the demonstration was continued all along the route to Clarence House. On Tuesday evening the Duke and Duchess left London for Dublin.

### Occupation of Sokoto.

It was announced in the House of Commons, on Monday afternoon, that Sir Frederick Lugard had captured Sokoto. Mr. Chamberlain read a despatch from Sir Frederick, dated Sokoto, March 19th, in which he stated that the place was occupied on March 15th, "after feeble resistance." The Sultan and chiefs fled. Sir Frederick hopes "to effect a full settlement early," and is breaking up the expeditionary force. This means that the military operations, which commenced on January 29, when Colonel Morland left the advanced post of Zaria for Kano, may be regarded as at an end, and that within about twelve months almost the whole of the vast area in the northern portion of Nigeria, with a frontier of something like a thousand miles, has been traversed by British forces and brought under effective control. Sir Frederick Lugard, who has been suffering from fever, is expected to reach home in May.

### General Sir Hector Macdonald.

The suicide, last week, of General Sir Hector Macdonald, Commander of the Forces in Ceylon, in a Paris hotel, has caused a most painful sensation. General Macdonald came to England a few weeks ago in order to consult the Commander-in-Chief with reference to the course he should pursue concerning certain grave charges brought against him, and it was announced that, acting upon the advice of Lord Roberts, he would return to Ceylon and demand a trial by court-martial. Instead of this, however, he went to Paris spent several days there, and ultimately shot himself in his room at the Hotel Regina. Sir Hector Macdonald was born on the 4th of March, 1853. He was a native of Urquhart, in Ross-shire, and was the youngest of five sons of a crofter and stonemason. At 15 he was apprenticed to a draper in Inverness, but in June, 1870, he left his employer's service and enlisted in the 92nd Regiment, now the 2nd Battalion of the Gordon Highlanders. He was soon afterwards sent out to India, and in 1879 he won distinction on the battlefield in the Afghan War. At Charasiah the young sergeant, as he then was, earned the medal with three clasps and the bronze star. Subsequently, at the instance of Lord Roberts, he received a commission, and was one of the officers in charge of the detachment which in 1881 took part in the fight on Majuba Hill. He was taken prisoner, but General Joubert was so impressed with the bravery of his defence that, on his release, he returned his sword to him. In Egypt he was yet more brilliantly successful, covering himself with military glory, afterwards, on his return to England, being appointed A.D.C. to Queen Victoria. In 1899 he went to India, and, on the death of General Wauchope, was called to the command of the Highland Brigade in South Africa. He led them to victory at Paardeberg, but was himself wounded. His services were rewarded by a K.C.B., and in 1901 he was appointed to the command in Ceylon. His remains were conveyed from Paris through London to Edinburgh, and laid to rest in the Dean Cemetery, Edinburgh, on Monday.

### Mr. Gladstone's Statue.

The statue of the late Mr. Gladstone, which Parliament authorised to be placed in Westminster Abbey, and which has been executed by Mr. Brock, R.A., was erected last week under the direction of the sculptor. On Sunday it was the object of general interest. It is situated in a prominent position on the east side of the north transept, close to the statues of Sir Robert Peel and Lord Beaconsfield. Like those monuments, it is of white marble, and has for its foundation a marble pedestal. Mr. Gladstone is represented standing, wearing the gown of a D.C.L. of Oxford University, and with his face turned slightly to the left toward the choir. The site was marked out many years ago by Dean Stanley as reserved for this particular purpose, and it is the last vacant position for a standing figure in the transept.

### A Book and its Story.

MRS. HUMPHRY WARD'S NEW NOVEL.*

Neither politics nor religion figure conspicuously in Mrs. Humphry Ward's new novel, "Lady Rose's Daughter," which is briskly and charmingly written, without one dull patch in the picture it gives of the men and women who formed the cultured circle of smart society by which the heroine was surrounded. Julie le Breton is the daughter of Lady Rose Delaney—once Lady Rose Chantrey—who had married Colonel Delaney. "They were not a happy couple—she was a woman of great intelligence, but endowed with one of those natures, sensitive, plastic, eager to search out and to challenge life which bring to their possessors some great joys hardly to be balanced against a final sum of pain. Her husband absorbed in his military life, silent, narrowly able . . . soon found her a tiring and trying companion. . . . Then there came on the scene a man of good family, thirty-five or so, traveller, painter, dreamer, . . . bringing with him the reputation of having plotted and fought for most of the 'lost causes' of our generation, including several which brought him into conflict with our British authorities and British officials." Marriott Dalrymple with his free and generous nature, his passion for things of the mind, at once filled all the vacant and unsatisfied spaces in the mind and heart of Lady Rose. "She fell in love with an intensity befitting her true temperament."

Within a short time of their meeting she had left her husband. Marriott Dalrymple returned her love, and the love of two people meant for each other was in his eyes strong enough to dispense with what he considered a mere legality. The pair severed themselves from England and retired to Belgium, where they rented a small eighteenth-century chateau some thirty miles from Brussels. It stood amid flat unpicturesque surroundings, lines of poplars outlined the meadows, and a field of beetroot grew up to its very walls, accentuating the shabby stateliness of its architecture. Here Julie was born, and Marriott Dalrymple and Lady Rose lived, for some years. He had been called an agitator, a leader of lost causes, and Lady Rose's small fortune had grown smaller from the demands which the claims of these causes, of many kinds, and in many countries, had made upon it. She survived Dalrymple ten years, years passed in poverty and seclusion in Bruges. Julie was sent to an Ursuline convent school, and upon her mother's death was confided to the care of her old *gouvernante*, Madame le Breton, whose name she took. Her grandfather, Lord Lackington, through his solicitor, allowed her a hundred a year on the understanding that no further claim was to be made on him, and that her mother's history was to die with her. Julie grew to maturity in Bruges. She was a source of considerable anxiety to the good nuns. Clever, too clever for their teaching, she was a born sceptic, notwithstanding that she was, after her mother's death, baptised into the Catholic Church. "Whenever she appeared she produced parties and the passions of parties. And though, as she grew older she showed much adroitness in managing those who were hostile to her, she was never without enemies, and intrigues followed her." In spite of these dangerous traits she had the power of drawing and attracting to herself some friends. There were English girls at Bruges belonging to an old Catholic family; and she, at their persuasion, had returned to England as their quasi-holiday governess. Thus she was first brought to the notice of Lady Henry Delafield, whose failing eyesight made it necessary for her to have a companion. She had been a leader of society, had held, and still wished to hold, a salon, but the infirmities of failing health and years were telling, and she realised unwillingly, that if she was to continue she must have someone who would help to receive, and retain around her, the circle which met weekly at her house. Lady Henry's comments on Julie, and her own situation at the moment, are offered to an old friend, Sir Wilfred Bury, who expresses sympathy. "Oh don't pity me! I don't pity other people. This odious body of ours has got to wear out some time—it's in the bargain. There are two things I care about; one is talk with the people that amuse me, and the other is the reading of French books." These two requirements Julie was eminently fitted to fulfil, and she was at once engaged, with the result, continues Lady Henry, that "everyone was amazed at her manners, her intelligence. She was perfectly modest—perfectly well-behaved. The old Duke —— was charmed with her, Montresor, Meredith, Lord Robert, all my *habitués* congratulated me. Such cultivation, such charm, such *savoir faire*." But as time went on Lady Henry became jealously suspicious of her protégée who was a centre of attraction to her guests. Julie made many friends in the new sphere in which she found herself, one which brought into play the diplomatic gifts which were natural to her. She held a small court of her own. She had many men allies and some devoted lovers. Perhaps Lady Henry's fear of rivalry was not without foundation, although the planes upon which they stood were as opposite as youth, and advanced years, could make them. Sir Wilfred Bury goes, soon after his return from abroad, to an evening reception in Lady Henry's drawing-room. Julie was accompanied by the fascinating Duchess of Crowborough. "Sir Wilfred watched the progress of Mademoiselle le Breton through the room. Wherever she moved she was met with smiles, deference, an eager attention. Here and there she made an introduction; she distributed a group; she moved a chair. . . . It was evident that her rule was at once absolute and welcome. Presently, when she accepted a seat, Sir Wilfred perceived, in the intervals of his conversation, that she became the leader of the most animated circle in the room . . . . man after man joined the group that stood or sat around her . . . . and in the centre of it, the brilliance of her black head, sharply seen against a background of rose brocade, the grace of her tall form which was thin almost to emaciation, the expressiveness of her strange features, the animation of her gestures, the sweetness of her voice, drew the eyes and the ears of half the room to Lady Henry's companion." But her position was becoming intolerable, and she resigned it. Her little friend, the Duchess, stood by her and promised the loan of a small furnished house, when she left Lady Henry. "Julie, dear Julie!" implored the Duchess. It's such a tiny little place, and it's quite musty for want of living in. Nobody has set foot in it for more than two years, and it would really be a kindness to us to go and live there. And there's the furniture just as it was, down to the bellows and snuffers! If you'd only use it and take care of it. Oh! do say yes, Julie." So Julie accepted the offer, and sent for Madame le Breton and her invalid daughter to act as friend and attendant. Left to live her own life she has time for the exercise of her many talents, and takes up literature, among other things, as a recreation. There are lovers and there is love in her life, but she, "like a woman," loves a man who is inferior to her, not socially, but in other ways. She uses her influence and gets him the command of an important mission abroad, which is to add to other distinctions already won. It is not our part to give her story away, but from this point it increases in dramatic interest and is brought to an artistic conclusion.

* "Lady Rose's Daughter." By Mrs. Humphry Ward. (1 vol. 6s. Smith Elder and Co.)

### For Reading to the Sick.

"I AM THY STAFF."

'O LORD, my God, the way is rough and long;
And I through weariness am faint and failing.'
"I am thy Staff, and I will strengthen thee,
Though earthly help is vain and unavailing."

'There is no water in this weary land,
While thirst consumes my parched and fainting Soul.'
"Come unto Me! of living Streams the Fount;
I will refresh thee; I will make thee whole."

'But, O my Lord! my heavy daily Cross
Doth well-nigh weigh me down. Lord, succour me!'
"I bear it with thee, O faint-hearted One,
Who a far heavier Cross have borne for thee.

"Fold not the darkness fondly round thy heart,
Think of My Mercy sweet, and comfort thee,
My poor, unworthy Child; for Mine thou art,
And sin alone can snatch My Child from Me."

*Elia.*

Jesu our Master, do Thou meet us while we walk by the way and long to reach the dear country; so that, following Thy light, we may keep the way of righteousness and never wander away into the dread darkness of this world's night; while Thou, Who art the Way, the Truth, and the Life, art shining within us.

O Christ, our God and our hope, we earnestly pray, implore and beseech Thee that, walking by Thy help, we may come unto Thee and rest in Thee. For Thou art the Way, the Truth, and the Life, without Whom no man cometh unto the Father.—*Anon.*

Our Lord's prayer, to be fulfilled in the day of His Glory, is that we may be, in the utmost fulness of its possible accomplishment, "one" with Him, as "Thou, Father, art in Me, and I in Thee, that they also may be one in Us, that the world may believe that Thou has sent Me. And the glory which Thou gavest me, I have given them; that they may be one, even as We are One." Within that transcendent circle of rest will the rest of the faithful be for ever. The unity of the Father and the Son with the Spirit, is the Bosom of Rest, within which enfolded the accepted members of the Son enter, as the result and proof of His acceptableness, which embraces not Himself only, but all who are His. Behold the repose of our wearied natures, the haven of the voyage over this troubled sea, the dwelling-place of the wanderer after his return, and his reception in his true Home!

Help us, O most Merciful! add to all Thy other gifts this further grace, without which all would be as nothing, that we may have increasing capacity to receive Thee, steadfastness to follow Thee, feeding on the opening Vision, till at last we shall see Thee, unveiled, face to face; still for a little while hidden, but at length seen in Thy fulness of Glory, and ourselves made for ever one with Thee, as Thou art One with the Father, in the light that can never fade, never fail us.

*T. T. Carter.*

We dare not deem that Heaven is dark,
Though Heaven's light seem dim;
Our Master looks upon us still
Although we see not Him;
He leads us onward in His Love,
He bears us in His Pity
To where the open Vision shines
In the Eternal City.

*R. S. J. A.*

### Notes and Queries.

**The Editor is always willing to answer in this column, without any fee, all reasonable questions, as soon as possible.**

**But the following rules must be carefully observed:—**

**1. Every communication must be accompanied by the name and address of the writer.**

**2. The question must always bear upon nursing, directly or indirectly.**

**If an answer is required by letter a fee of half-a-crown must be enclosed with the note containing the inquiry, and we cannot undertake to forward letters addressed to correspondents making inquiries. It is therefore requested that our readers will not enclose either a stamp or a stamped envelope.**

*Spina Bifida.*

(1) Is Spina Bifida common amongst new-born children, and is it usual for the child to have hydrocephalus as well?—*Bessie.*

Spina Bifida is not common. When met with there is frequently hydrocephalus present as well.

*Light-Therapeutics.*

(2) I should esteem it a favour if any of your scientific correspondents can inform me as to the action of the red rays of light on the hair and scalp, and what authority there is for supposing it to be beneficial.

Red rays are less chemically active than other light rays; it is for this reason that they are useful in photography, and for a similar reason they most probably exert less action on the hair and scalp than any other light rays.

*Poisoning by Carbolic Acid.*

(3) I should be glad if you will give me the name of the best emetic to administer in a case of poisoning by carbolic acid whilst waiting for the doctor's arrival?—*Nurse S.*

The best emetic to use is made by adding two tablespoonfuls of common salt to 8 ounces of warm water.

*Epigastrical Hernia.*

(4) Will you kindly tell me if an operation for an epigastrical hernia can be safely cured, and how is the case nursed?—*Sister Alice.*

This is a question for a medical man. The nursing is similar to that required after any other hernia operation; to answer this part of your question would fill too much space here.

*How to Humanise Milk.*

(5) I should be glad to know how to humanise milk, or where I could obtain a recipe. It is important to get this recipe, as it is wanted for a lady who is living in an out-of-the-way place in the tropics.—*Maternity Nurse.*

Take pure new milk, 6 teaspoonfuls; cream, 4 teaspoonfuls; Demerara sugar, 2 teaspoonfuls; barley water (freshly made), 2½ ozs. Pour the mixture into a bottle, the neck of which is then plugged with cotton-wool. Place it in a saucepan of warm water and raise to boiling; keep it at the boil for 30 minutes, and then let it cool. This is only applicable to cow's milk. The lady had better make sure that fresh cow's milk can be obtained in that part of the tropics to which she is going.

*The Midwives' Board.*

(6) Will you kindly tell me to whom and where I should address a communication for the Midwives' Board?—*C. J.*

At present the Board has no office, and all communications should be addressed to the Chairman, Midwives' Board, Privy Council Office, Whitehall, S.W.

*Books.*

(7) Will you kindly tell me the name of a good book on surgical nursing, about 4s. or 5s.?—*Faith.*

"Surgical Ward Work," by Alexander Miles, M.D. Scientific Press, 28 Southampton Street, Strand, W.C., price 3s. 10d. post free.

**Important Nursing Textbooks.**

"The Nursing Profession: How and where to Train." 2s. net; 2s. 4d. post free.

"A Handbook for Nurses." (New Edition). 5s. net; 5s. 4d. post free.

"The Human Body." 5s. post free.

"Ophthalmic Nursing." (New Edition). 3s. 6d. net; 3s. 10d. post free.

"Gynæcological Nursing." 1s. post free.

"Art of Feeding the Invalid." (Popular Edition). 1s. 6d. post free.

"Practical Hints on District Nursing." 1s. post free.

## Figures and Tables

**Figure f1:**
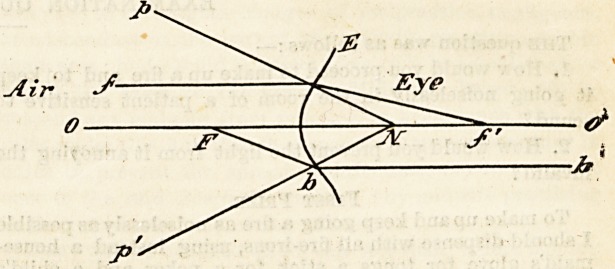


**Figure f2:**